# Timing of Evolocumab Initiation After Acute Coronary Syndrome and Long-Term Lipid and Cardiovascular Outcomes: A Multicenter Real-World Cohort Study

**DOI:** 10.3390/ph19071035

**Published:** 2026-07-02

**Authors:** Lama Alfehaid, Ehsan A. Habeeb, Amal M. Badawoud, Salwa A. Alsuhaibani, Rasha Almutairi, Rafeef Alyahya, Shoug Alquraishi, Hanin Alharbi, Sara Alshammari, Hanan Alfulayyih, Waad Almasoud, Ali A. Almakrami, Abdulaali Almutairi, Majed S. Al Yami

**Affiliations:** 1 Department of Pharmacy Practice, College of Pharmacy, King Saud bin Abdulaziz University for Health Sciences, Riyadh 14611, Saudi Arabia; 2 King Abdullah International Medical Research Center, Riyadh 11481, Saudi Arabia; 3 Pharmaceutical Care Department, King Abdulaziz Medical City, Riyadh 14611, Saudi Arabia; 4 Department of Pharmacy Practice, College of Pharmacy, Taibah University, Madinah 42353, Saudi Arabia; 5 Prince Mohammad Bin Abdulaziz Hospital, National Guard Health Affairs, Madinah 42324, Saudi Arabia; 6 Department of Pharmacy Practice, College of Pharmacy, Princess Nourah Bint Abdulrahman University, Riyadh 11671, Saudi Arabia; 7 Prince Sultan Cardiac Center, Qassim Health Cluster, Ministry of Health, Buraydah 52366, Saudi Arabia; 8 College of Pharmacy, Qassim University, Buraydah 52571, Saudi Arabia; 9 Clinical Pharmacy Department, King Fahad Medical City, Riyadh 12231, Saudi Arabia; 10 Department of Pharmacy Practice, College of Pharmacy, Alfaisal University, Riyadh 11533, Saudi Arabia

**Keywords:** evolocumab, acute coronary syndrome, atherosclerotic cardiovascular disease, secondary prevention, LDL cholesterol, PCSK9 inhibitors, real-world evidence, lipid-lowering therapy, cardiovascular outcomes, timing of initiation

## Abstract

**Background**: Intensive low-density lipoprotein cholesterol (LDL-C) reduction is essential for secondary prevention after acute coronary syndrome (ACS). Although randomized trials support the use of proprotein convertase subtilisin/kexin type 9 (PCSK9) inhibitors, real-world evidence on long-term lipid control and the optimal timing of initiation remains limited. **Objective**: To evaluate lipid outcomes and atherosclerotic cardiovascular disease (ASCVD) events associated with evolocumab after ACS and to assess whether timing of initiation influences these outcomes in routine clinical practice. **Methods**: This multicenter, retrospective cohort study included adults who were initiated on evolocumab following ACS between 2017 and 2024. Lipid parameters were assessed at predefined follow-up time points (3 months, 6 months, 1 year, 2 years, and up to 3 years) using an available-case approach. Patients were categorized as early initiators (≤1 month) or late initiators (>1 month). Recurrent ASCVD events, hospitalization burden, and mortality were analyzed using multivariable regression. A propensity score-matched sensitivity analysis was also performed. **Results**: Among 525 included patients (mean age 53.1 ± 11.6 years; 80.2% male), baseline LDL-C was 3.68 ± 1.66 mmol/L. LDL-C decreased to approximately 1.8–2.0 mmol/L within 3 months, corresponding to an approximate 45–50% reduction from baseline, with consistent reductions observed across available follow-up time points. Recurrent ASCVD events occurred in 14.8% of patients, and in-hospital mortality was 2.3%. Although early initiators had higher baseline risk, adjusted analyses showed no statistically significant association between early initiation and recurrent ASCVD (adjusted OR 1.50; 95% CI 0.82–2.70; *p* = 0.17). Similarly, no statistically significant differences in lipid outcomes were observed between early and late initiation groups after adjustment. Findings were consistent in propensity score-matched analyses. **Conclusions**: In this real-world post-ACS cohort, evolocumab was associated with substantial and sustained LDL-C reduction across follow-up time points. No significant associations were observed between timing of initiation and lipid or ASCVD outcomes after adjustment. These findings should be interpreted cautiously, given the observational design, available-case analysis, and potential for residual confounding.

## 1. Introduction

Lowering low-density lipoprotein cholesterol (LDL-C) remains a cornerstone of secondary prevention in patients with established atherosclerotic cardiovascular disease (ASCVD). Numerous studies have demonstrated a continuous relationship between LDL-C reduction and cardiovascular risk, with greater reductions associated with fewer recurrent cardiovascular events. Consequently, contemporary guidelines emphasize both substantial relative LDL-C lowering (≥50% from baseline) and achievement of low absolute LDL-C targets, particularly among patients at very high cardiovascular risk [1,2,3].

The 2019 European Society of Cardiology/European Atherosclerosis Society (ESC/EAS) guidelines recommend LDL-C levels below 55 mg/dL (<1.4 mmol/L) for very high-risk patients and below 40 mg/dL (<1.0 mmol/L) for selected patients with recurrent ASCVD events [1]. More recently, the 2025 ESC focused update further emphasized the importance of achieving LDL-C targets as early as possible after acute coronary syndrome (ACS), supporting earlier treatment intensification, broader use of combination lipid-lowering therapy, and consideration of proprotein convertase subtilisin/kexin type 9 (PCSK9) inhibitors in patients unlikely to achieve recommended targets with statin-based therapy alone [2]. Similarly, the 2025 ACC/AHA multisociety guideline advocates intensive lipid-lowering strategies based on overall cardiovascular risk and cumulative exposure to atherogenic lipoproteins, reflecting an increasing focus on earlier risk reduction following ACS [3].

Despite these recommendations, attainment of guideline-recommended LDL-C targets remains suboptimal in routine clinical practice. High baseline LDL-C levels, inadequate response to conventional lipid-lowering therapy, medication intolerance, and challenges related to long-term adherence frequently necessitate the addition of non-statin therapies, including ezetimibe and PCSK9 inhibitors [4,5,6].

Randomized clinical trials have established the efficacy of PCSK9 inhibitors in achieving substantial LDL-C reductions and improving cardiovascular outcomes among high-risk patients [7,8,9,10]. More recently, the VESALIUS-CV trial demonstrated that evolocumab significantly reduced major adverse cardiovascular events in high-risk patients without a prior myocardial infarction or stroke who were receiving optimized lipid-lowering therapy, extending the evidence base for intensive LDL-C lowering beyond traditional secondary prevention populations [11]. Collectively, these findings support the concept that earlier and more intensive LDL-C lowering may reduce cumulative exposure to atherogenic lipoproteins and improve long-term cardiovascular outcomes.

While the benefits of intensive LDL-C reduction are well established, the optimal timing of PCSK9 inhibitor initiation following ACS remains less clearly defined in routine clinical practice. Treatment initiation is influenced by multiple factors, including baseline cardiovascular risk, LDL-C levels, clinician judgment, healthcare system processes, medication access, and patient preferences. Consequently, substantial variability exists in the timing of PCSK9 inhibitor implementation after ACS. Moreover, real-world evidence evaluating whether earlier initiation is associated with improved long-term lipid control or cardiovascular outcomes remains limited.

To address this knowledge gap, we conducted a multicenter, real-world cohort study evaluating the use of evolocumab following ACS within a large Saudi healthcare system. The objectives were to characterize long-term lipid responses, compare lipid responses with early versus later evolocumab initiation, and explore associated ASCVD outcomes, including recurrent ASCVD events, hospitalization burden, and mortality. Given the observational nature of the study and the potential for treatment-selection bias, these analyses were intended to characterize real-world practice patterns and generate hypotheses rather than establish causal relationships.

## 2. Results

### 2.1. Baseline Characteristics

A total of 525 patients were included (mean age 53.1 ± 11.6 years; 80.2% male). Cardiometabolic disease burden was high, with diabetes present in 68.6%, hypertension in 72.7%, dyslipidemia in 92.0%, and established ASCVD in 50.0% of the cohort. ASCVD risk-equivalent conditions included metabolic syndrome (22.3%), elevated triglycerides (21.3%), LDL-C ≥190 mg/dL (13.0%), and familial hypercholesterolemia (5.4%).

Most patients were receiving guideline-recommended preventive therapy prior to ACS, including aspirin (88.6%), beta-blockers (86.9%), high-intensity statins (68.8%), and ezetimibe (13.6%). During follow-up after initiation of evolocumab, 466 patients (88.8%) remained on background statin therapy, while 222 (42.3%) received ezetimibe. Few patients (4.8%) reported a history of statin intolerance before ASCVD events. Baseline patient characteristics are summarized in Table 1.

### 2.2. Lipid Profile Changes from Baseline Across Follow-Up Time Points

Baseline lipid values prior to evolocumab initiation are presented alongside follow-up measurements in Table 2. Mean baseline LDL-C was 3.68 ± 1.66 mmol/L, with reductions to approximately 1.8–2.0 mmol/L observed within 3 months of therapy initiation, corresponding to an approximate 45–50% reduction from baseline. LDL-C levels remained relatively stable across subsequent follow-up time points, with a mean of 2.01 mmol/L at 3 years.

Similar patterns were observed for total cholesterol (TC), which decreased substantially from baseline and remained lower across follow-up. Triglycerides (TG) demonstrated modest reductions, primarily during the first year, while high-density lipoprotein cholesterol (HDL-C levels remained largely unchanged throughout the observation period.

Overall, consistent lipid reductions were observed across available follow-up time points (Table 2). However, the number of patients contributing to each time point varied, reflecting real-world follow-up patterns and the availability of laboratory measurements rather than a fixed longitudinal cohort.

### 2.3. Lipid Changes in Patients with Recurrent ASCVD

A total of 77 patients (14.8%) experienced recurrent ASCVD events. Among these patients, LDL-C and TC levels decreased across all evaluated time points. Mean reductions in LDL-C ranged from −1.57 mmol/L at 3 months to −1.10 mmol/L at 3 years (all *p* < 0.001), with similar reductions observed for total cholesterol.

TG demonstrated modest reductions during the first year, but these were not significantly different from baseline at later time points. HDL-C levels remained largely unchanged throughout follow-up.

These findings suggest that lipid-lowering effects were maintained in patients who experienced recurrent ASCVD events; however, these observations should be interpreted descriptively given the high baseline risk in this subgroup. The number of patients contributing to later time points decreased (n = 36 at 3 years), reflecting variability in real-world follow-up and laboratory availability rather than solely loss to follow-up (Table 3).

### 2.4. Comparison Between Early vs. Late Evolocumab Initiation

Of 511 evaluable patients, 155 (30.3%) initiated evolocumab early and 356 (69.7%) initiated therapy later. Early initiators had higher baseline LDL-C (4.08 vs. 3.51 mmol/L) and TC (5.61 vs. 5.03 mmol/L), whereas late initiators had a greater burden of cardiometabolic comorbidities, including diabetes mellitus, hypertension, long-standing diabetes, and metabolic syndrome.

During follow-up, most patients in both groups remained on background statin therapy, although continuation was more frequent among late initiators (94.6% vs. 80.0%; *p* < 0.001). Among patients with documented dosing, high-intensity statins were the predominant regimen in both groups (61.5% vs. 56.8%; *p* = 0.006). Moderate-intensity therapy was used in approximately one-quarter of patients, while low-intensity therapy was uncommon. Ezetimibe use was similar between groups (44.9% vs. 38.7%; *p* = 0.226) (Table 4).

These baseline and treatment differences highlight the potential for confounding by indication in comparisons between early- and late-initiation groups.

**Figure 1:**
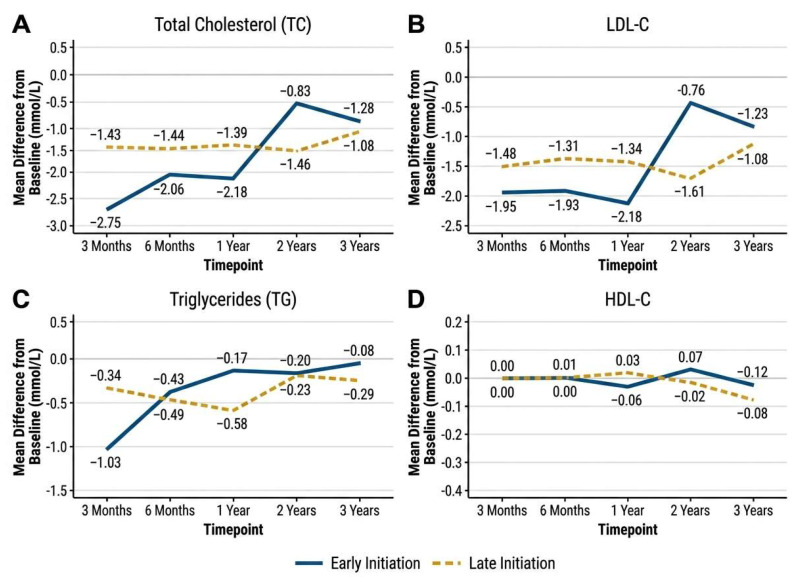
Mean Changes in Lipid Parameters Across Follow-up Time Points by Timing of Evolocumab Initiation. Mean changes from baseline in (**A**) total cholesterol (TC), (**B**) LDL-C, (**C**) triglycerides (TG), and (**D**) HDL-C among patients with early (≤1 month) and late (>1 month) evolocumab initiation after ACS. Values represent mean change from baseline (follow-up minus baseline). Analyses were performed using an available-case approach; therefore, patient numbers varied across time points. Abbreviations: ACS, acute coronary syndrome; HDL-C, high-density lipoprotein cholesterol; LDL-C, low-density lipoprotein cholesterol; TC, total cholesterol; TG, triglycerides.

Despite these baseline and treatment differences, both groups achieved substantial reductions in LDL-C and total cholesterol across follow-up time points. Early initiators demonstrated greater unadjusted lipid reductions, likely reflecting their higher baseline lipid levels (Figure 1). However, after adjustment for baseline characteristics, no statistically significant differences were observed between early and late initiation groups at any follow-up time point for LDL-C, TC, TG, or HDL-C. Overall, lipid changes were comparable between groups regardless of the timing of evolocumab initiation (Table 5).

### 2.5. Adjusted vs. Unadjusted Differences in Lipid Changes

In unadjusted analyses, early initiators appeared to achieve greater reductions in LDL-C and total cholesterol during the first year. However, these differences were attenuated after adjustment for baseline characteristics, including initial lipid levels and comorbidities.

Across follow-up intervals, no consistent or statistically significant differences were observed between early and late initiation groups in adjusted analyses. HDL-C remained unchanged in both groups. These findings suggest that the observed differences in unadjusted analyses were largely attributable to baseline imbalances rather than to the timing of initiation (Table 5).

### 2.6. ASCVD Outcomes

During follow-up, recurrent ASCVD events occurred in 14.8% of patients, most commonly non-ST-segment elevation myocardial infarction (6.5%), unstable angina (3.2%), and ST-segment elevation myocardial infarction (2.1%). In-hospital mortality was 2.3%.

Recurrent ASCVD events occurred in 18.4% of early initiators compared with 13.2% of late initiators (*p* = 0.171). After multivariable adjustment, early initiation was not significantly associated with recurrent ASCVD events (adjusted OR 1.50, 95% CI 0.82–2.70; *p* = 0.170). Rates of repeat coronary revascularization were numerically higher among early initiators (17.1% vs. 11.1%), although this difference was not statistically significant (*p* = 0.240). Similarly, second recurrent ASCVD events occurred more frequently among early initiators (6.2% vs. 3.8%; *p* = 0.385).

Early initiators were more likely to experience ≥2 ASCVD-related hospitalizations (25.5% vs. 13.4%; *p* < 0.001), whereas a greater proportion of late initiators had no ASCVD-related hospitalizations during follow-up (38.4% vs. 20.9%; *p* < 0.001). In-hospital mortality remained low and was similar between groups (2.6% vs. 2.0%; *p* = 0.916) (Table 5).

These findings should be interpreted in the context of baseline differences between groups and the potential for confounding by indication.

Values are presented as mean ± standard deviation or number (percentage), unless otherwise specified. Lipid parameters were assessed at predefined follow-up time points using an available-case approach; therefore, the number of patients contributing to each time point varied according to laboratory availability and follow-up duration. Early initiation was defined as evolocumab initiation ≤ 1 month after the index ACS event, whereas late initiation was defined as initiation > 1 month after the index ACS event. *p*-values for unadjusted comparisons were calculated using independent *t*-tests for continuous variables and χ^2^ tests for categorical variables. Adjusted lipid outcome analyses were performed using multivariable linear regression models accounting for baseline demographic characteristics, lipid values, and ASCVD risk factors.

### 2.7. Propensity Score-Matched Sensitivity Analysis

#### 2.7.1. Matched Cohort Characteristics

To further evaluate the association between timing of evolocumab initiation and outcomes while minimizing baseline imbalance, a propensity score-matched analysis was performed. Following 1:1 nearest-neighbor matching, 80 matched pairs were identified.

Covariate balance improved substantially after matching, with a mean standardized mean difference (SMD) of 0.032 and a maximum SMD of 0.172. No covariate exceeded the predefined threshold of SMD > 0.20 after matching, indicating an acceptable balance between groups.

#### 2.7.2. Lipid, Hospitalization, and Recurrent ASCVD Count Outcomes in the Matched Cohort

Linear regression analyses were performed within the matched cohort to compare lipid parameters between early and late evolocumab initiation groups across follow-up time points.

No statistically significant differences were found between the groups for LDL-C, total cholesterol, HDL-C, or triglycerides at 3 months, 6 months, 1 year, or 2 years. While early initiators showed numerically lower LDL-C and TC at some earlier points, these differences were not statistically significant after matching. Conversely, early initiators had a numerically higher TC level at 2 years, but this difference did not reach statistical significance (*p* = 0.052).

The 3-year follow-up interval was excluded from the regression analyses due to insufficient sample size.

The number of ASCVD-related hospitalizations was numerically higher among early initiators (β = 0.325, 95% CI −0.001 to 0.651; *p* = 0.051), although this difference did not reach statistical significance. No significant difference in the total number of recurrent ASCVD events was observed between groups.

Overall, the matched analysis demonstrated comparable outcomes regardless of the timing of evolocumab initiation (Table 6).

All lipid values are reported in mmol/L. Regression analyses were performed in the propensity score-matched cohort. The reference group was the late initiation group. Sample sizes varied across follow-up time points because analyses were conducted using an available-case approach based on available laboratory and clinical data at each interval. The 3-year lipid analysis was not performed because of insufficient sample size.

#### 2.7.3. ASCVD Outcomes in the Matched Cohort

Binary logistic regression analyses in the propensity score-matched cohort demonstrated no statistically significant differences in recurrent ASCVD outcomes between early and late evolocumab initiation groups. First recurrent ASCVD events occurred in 18.8% of early initiators compared with 15.0% of late initiators (OR 1.31, 95% CI 0.57–3.05; *p* = 0.527). Similarly, no statistically significant differences were observed for second recurrent ASCVD events (11.1% vs. 7.4%; OR 1.58, 95% CI 0.48–5.59; *p* = 0.459) or third recurrent ASCVD events (6.5% vs. 4.5%; OR 1.45, 95% CI 0.31–7.61; *p* = 0.637).

Logistic regression analyses for recurrent ASCVD outcomes in the matched cohort are summarized in Table 7.

Logistic regression analyses were performed in the propensity score-matched cohort. Sample sizes varied across outcomes because analyses were conducted using available-case data based on event availability and follow-up documentation. The reference group was the late initiation group. OR >1 indicates higher odds in the early initiation group. Recurrent ASCVD events included non-ST-segment elevation myocardial infarction, ST-segment elevation myocardial infarction, unstable angina, ischemic stroke, sudden cardiac death, and repeat coronary revascularization.

## 3. Discussion

This multicenter, retrospective, real-world study evaluated the effectiveness of evolocumab in Saudi patients following ACS, with the primary objective of characterizing prescribing patterns and assessing lipid responses and ASCVD outcomes in routine clinical practice. Conducted across multiple tertiary care centers, the analysis demonstrated that evolocumab use was associated with substantial and sustained reductions in LDL-C and TC over up to three years of follow-up. LDL-C levels declined to approximately 1.8–2.0 mmol/L as early as 3 months after initiation and remained stable through year 3, indicating durable lipid-lowering effectiveness outside the controlled environment of randomized clinical trials. Similar lipid-lowering patterns, including marked reductions in LDL-C and TC with minimal changes in HDL-C, were observed among patients who experienced recurrent ASCVD events, suggesting preserved pharmacologic effectiveness even in persistently high-risk patients.

Despite differences in baseline risk profiles, including a higher cardiometabolic burden among late initiators and higher baseline LDL-C levels among early initiators, both groups achieved comparable reductions in lipid levels after multivariable adjustment. No statistically significant differences in adjusted lipid outcomes were observed between groups. Recurrent ASCVD events occurred in 14.8% of patients, while in-hospital mortality remained low (2.3%), reflecting the substantial residual ASCVD risk in this high-risk post-ACS population.

An important observation in this study was the numerically higher recurrence rate and hospitalization burden among early initiators. In routine clinical practice, earlier intensification with PCSK9 inhibitors is often preferentially reserved for patients perceived to be at particularly high ASCVD risk, including those with markedly elevated LDL-C levels or more aggressive disease phenotypes. In our cohort, early initiators had significantly higher baseline LDL-C levels, suggesting more severe underlying dyslipidemia and potentially greater atherosclerotic burden at presentation. Although multivariable adjustment and propensity score matching substantially improved measured baseline balance, residual confounding and confounding by indication remain likely. Unmeasured factors, including ACS severity, coronary anatomy, inflammatory burden, completeness of revascularization, and treatment adherence, may have influenced both treatment timing and subsequent outcomes. Importantly, early initiation was not independently associated with recurrent ASCVD events after adjustment or propensity score matching, suggesting that the numerically higher event burden observed among early initiators likely reflects underlying baseline risk rather than a treatment-related effect.

The LDL-C reductions observed in this study align with phase 3 trial data demonstrating 50–60% LDL-C lowering with PCSK9 inhibitors [7,8,9]. In the FOURIER trial, evolocumab produced a 59% reduction in LDL-C, achieving a median on-treatment LDL-C of 30 mg/dL (0.78 mmol/L) [9]. Similarly, the ODYSSEY OUTCOMES trial demonstrated substantial LDL-C reductions and significant ASCVD benefits with alirocumab in post-ACS populations [10]. Although absolute LDL-C levels in our real-world cohort remained higher than those reported in randomized trials, this difference is expected given baseline variability, treatment heterogeneity, adherence challenges, and non-standardized laboratory follow-up in routine care. Nevertheless, the direction and magnitude of lipid reduction remained consistent with the established effectiveness of PCSK9 inhibitors.

Real-world evidence from Middle Eastern and global practice further supports these findings. The ZERBINI study, conducted in Saudi Arabia and Kuwait, demonstrated an approximate 58% reduction in LDL-C with evolocumab and high treatment persistence [12]. Similarly, pooled phase 3 analyses and systematic reviews have consistently demonstrated robust lipid-lowering efficacy and favorable safety profiles for PCSK9 monoclonal antibodies across diverse populations [8]. Compared with prior studies, the present analysis adds value by evaluating a high-risk Saudi post-ACS population with extended follow-up and by directly examining early versus late initiation, an area that remains relatively underexplored in real-world PCSK9 inhibitor research.

The ASCVD outcomes observed in this study are also consistent with prior evidence. In FOURIER, recurrent ASCVD events occurred in 9.8% of patients over a median follow-up of 2.2 years [9], while ODYSSEY OUTCOMES demonstrated a 15% relative reduction in major adverse cardiovascular events after ACS [10]. The 14.8% recurrence rate observed in this cohort is therefore reasonable given the high prevalence of diabetes, metabolic syndrome, and other ASCVD risk equivalents. Unlike randomized trials, however, early initiation of evolocumab was not associated with a statistically significant reduction in recurrent events, likely reflecting residual confounding inherent to observational designs rather than the absence of therapeutic benefit.

Background LLT during follow-up was largely consistent with contemporary secondary prevention standards. Most patients remained on statin therapy after ACS and during evolocumab treatment, and high-intensity statins represented the predominant regimen in both groups. Although statin continuation was more frequent among late initiators, ezetimibe use and statin intensity were broadly comparable between groups, suggesting that major differences in lipid outcomes were unlikely to be driven solely by disparities in concomitant LLT. Nonetheless, real-world adherence, dose adjustments over time, and incomplete documentation remain potential sources of heterogeneity that could not be fully captured.

The definition of early initiation as ≤1 month after ACS was selected to reflect a clinically meaningful post-ACS treatment window consistent with guideline recommendations to reassess lipid levels and intensify therapy within approximately 4 weeks [1,2,3]. This cutoff was intended to reflect real-world prescribing patterns rather than a biologically discrete threshold. More granular categorization of treatment timing may provide additional insight; however, further subdivision within this retrospective cohort would substantially reduce subgroup sample sizes and statistical stability. Accordingly, comparisons between early and late initiation should be interpreted as exploratory and hypothesis-generating.

The findings reinforce the importance of integrating PCSK9 inhibitors into routine secondary prevention strategies for patients who fail to achieve LDL-C targets despite statin-based therapy. The durable lipid-lowering effect observed over three years indicates that evolocumab remains effective in real-world Saudi practice. Comparable lipid outcomes between early- and late-initiation groups suggest that treatment timing alone should not deter clinicians from introducing PCSK9 inhibitors later in the disease course when LDL-C goals remain unmet. At the same time, persistent recurrent ASCVD risk despite substantial LDL-C reduction highlights the need for comprehensive cardiovascular risk reduction strategies beyond lipid-lowering alone.

This study has several strengths, including its large multicenter design, extended follow-up, and comprehensive evaluation of lipid and ASCVD outcomes in a high-risk post-ACS population. The analysis also provides clinically relevant insight into the real-world implementation of evolocumab and contemporary guideline-directed LLT across Saudi tertiary care centers.

Several limitations should also be acknowledged. Laboratory assessments were not protocol-driven, and follow-up intervals varied substantially across patients. Analyses were therefore conducted using an available-case approach, resulting in partially overlapping patient subsets across time points rather than true longitudinal within-patient trajectories. Missingness increased at later follow-up intervals and was likely influenced by clinical stability, follow-up duration, and laboratory ordering practices, introducing potential informative missingness and survivor bias.

Although multivariable adjustment and propensity score matching were performed, residual confounding remains likely because treatment timing was determined by clinician judgment and underlying disease severity. The matched analysis also resulted in a reduced effective sample size and could not account for unmeasured confounding variables. Medication adherence, persistence, and changes in background LLT during follow-up could not be fully captured. Additionally, inflammatory and vascular remodeling markers such as the neutrophil-to-lymphocyte ratio (NLR) and carotid intima-media thickness (CIMT) were not routinely collected across participating centers and therefore could not be evaluated. Future prospective studies incorporating serial inflammatory biomarkers and imaging-based measures of atherosclerotic burden may provide additional mechanistic insight into the relationship between timing of PCSK9 inhibitor initiation, vascular remodeling, and long-term cardiovascular outcomes [13,14,15,16]. Additionally, ASCVD outcomes were analyzed using logistic regression rather than time-to-event methods because precise event timing was inconsistently available. Consequently, the reported estimates should be interpreted as cumulative risks within the observed follow-up period rather than time-dependent hazards. Finally, ASCVD outcomes were derived from electronic medical records and were not formally adjudicated using standardized definitions.

## 4. Materials and Methods

### 4.1. Study Design and Settings

This multicenter, retrospective cohort study was conducted through a comprehensive review of electronic medical records from King Fahad Medical City (KFMC) and National Guard Health Affairs hospitals, including King Abdulaziz Medical City (KAMC) in Riyadh, Jeddah, and Al-Ahsa, Saudi Arabia, as well as Prince Sultan Cardiac Center (PSCC) in Al-Qassim. The study period spanned from January 2017 to December 2024. Ethical approval was obtained from the Institutional Review Boards of the participating institutions, including King Fahad Medical City, Riyadh, Saudi Arabia (IRB No. 24-588), and the National Guard Health Affairs hospitals, Saudi Arabia (IRB No. 0000028824). The study was conducted in accordance with the Declaration of Helsinki.

Adult patients aged 18 years or older were eligible for inclusion if they were initiated on evolocumab following an ACS event and had received at least one documented dose of the medication. Patients were excluded if substantial portions of their clinical care were delivered outside the participating centers or if they were lost to follow-up during the study period.

Collected variables included demographic characteristics, clinical comorbidities, laboratory parameters, and pharmacologic therapies. Background lipid-lowering therapy, including statin use and intensity (high-, moderate-, or low-intensity where documented) and other non-statin agents, was recorded at baseline and during follow-up when available.

### 4.2. Study Outcomes and Follow-Up

The primary outcome of the study was the change in lipid parameters following initiation of evolocumab in routine clinical practice. Lipid parameters of interest included LDL-C, TC, TG, and HDL-C. Lipid values were obtained from routine laboratory measurements during follow-up and were assessed at predefined time points (approximately 3 months, 6 months, 1 year, 2 years, and up to 3 years after initiation of evolocumab). For each interval, the measurement closest to the target time point was selected.

Secondary outcomes focused on ASCVD events during follow-up. Recurrent ASCVD outcomes were defined as the occurrence of any subsequent ASCVD event after evolocumab initiation, including non-ST-segment elevation myocardial infarction, ST-segment elevation myocardial infarction, unstable angina, ischemic stroke, sudden cardiac death, or repeat coronary revascularization. The burden of ASCVD-related hospitalizations was also evaluated and categorized into none, 1, or 2 or more hospitalizations during the follow-up period. In-hospital mortality was defined as death occurring during an ASCVD-related hospitalization.

Additional analyses evaluated lipid changes across follow-up time points among patients who experienced recurrent ASCVD events despite ongoing LLT, to assess whether evolocumab maintained lipid-lowering effectiveness in this higher-risk subgroup. Lipid responses and ASCVD outcomes were also compared between patients with early initiation of evolocumab (≤1 month after the index ACS event) and those with late initiation (>1 month after the index ACS event) to describe real-world treatment patterns. The ≤1-month cutoff was selected to reflect a clinically relevant post-ACS treatment window consistent with guideline recommendations to reassess lipid levels and intensify therapy within approximately 4 weeks; however, this definition was intended to capture real-world prescribing patterns rather than to represent a biologically discrete threshold.

These comparisons were exploratory in nature and were not intended to support causal inference. Patients were followed from the time of evolocumab initiation until the occurrence of an ASCVD event, death, loss to follow-up, or the end of the study period, whichever occurred first.

### 4.3. Statistical Analysis

Descriptive statistics were used to summarize baseline demographic and clinical characteristics. Continuous variables are presented as means with standard deviations, and categorical variables as frequencies and percentages.

Lipid outcomes were evaluated at predefined follow-up time points (approximately 3, 6, 12, 24, and 36 months). For each interval, the measurement closest to the prespecified time point within a ±30-day window was selected. Analyses were conducted using an available-case approach; therefore, the number of patients contributing to each time point varied.

Comparisons between baseline and follow-up lipid values among patients with paired measurements available at each interval were performed using paired *t*-tests. Between-group comparisons (early vs. late initiation) were performed using independent *t*-tests for continuous variables and χ^2^ tests for categorical variables.

ASCVD-related hospitalization burden (none, 1, or ≥2 admissions) and in-hospital mortality were summarized descriptively and compared between groups using appropriate categorical tests. To account for baseline differences between early and late initiation groups, multivariable linear regression models were used to evaluate adjusted differences in lipid outcomes. Covariates were selected a priori based on clinical relevance and baseline imbalances, including demographic characteristics, baseline lipid levels, and established ASCVD risk factors.

Recurrent ASCVD events were analyzed using multivariable logistic regression. Because follow-up duration varied across patients and precise event timing was not consistently available, time-to-event analyses (e.g., Kaplan–Meier or Cox proportional hazards models) were not feasible. Accordingly, logistic regression was used to estimate associations within the observed follow-up period, and these estimates should be interpreted as cumulative risks rather than time-dependent hazards.

Mixed-effects models were considered; however, given the irregular follow-up intervals and incomplete repeated measurements, such models may yield unstable estimates and were therefore not applied.

A two-sided *p*-value < 0.05 was considered statistically significant. All analyses were conducted using R software (version 4.4.2; R Foundation for Statistical Computing, Vienna, Austria; https://www.r-project.org/; accessed on 20 September 2025).

### 4.4. Propensity Score Matching Analysis

To further address confounding by indication and baseline imbalances between early and late evolocumab initiation groups, a propensity score-matched sensitivity analysis was performed. Propensity scores were estimated using a generalized boosted model (GBM) implemented through the twang package in R, targeting the average treatment effect on the treated (ATT). The model incorporated 38 baseline covariates, including demographic characteristics, cardiovascular comorbidities, prior ASCVD history, lipid-lowering and cardiovascular therapies, and laboratory parameters. Variables with zero variance were excluded before model fitting.

The GBM was configured with 20,000 trees, an interaction depth of 3, and a shrinkage rate of 0.005. Optimal balance was determined using the mean standardized difference criterion, minimizing overall covariate imbalance without relying on parametric assumptions.

Patients were subsequently matched 1:1 without replacement using nearest-neighbor matching on the logit of the propensity score with a caliper width of 0.2 standard deviations (MatchIt package). Covariate balance before and after matching was assessed using standardized mean differences (SMDs), with SMD < 0.10 considered indicative of acceptable balance. Balance diagnostics, including Love plots and propensity score density distributions, were generated using the cobalt package.

Outcome analyses in the matched cohort included linear regression for continuous outcomes and logistic regression for binary outcomes. Results are presented as regression coefficients (β) or odds ratios (ORs) with 95% confidence intervals (CIs). Due to the limited sample size at the 3-year follow-up, this time point was excluded from regression analyses.

### 4.5. Sample Size Considerations

This was a retrospective cohort study including all eligible patients treated during the study period; therefore, a formal a priori sample size calculation was not performed. The study sample was determined by available real-world data across participating centers.

### 4.6. Missing Data

Given the observational design and non-standardized follow-up intervals, lipid measurements were not available for all patients at each time point. Analyses were conducted using available-case data without imputation. Patterns of missingness were evaluated descriptively and were likely influenced by variability in clinical follow-up and patient stability. Missing data were not assumed to be random, and informative missingness cannot be excluded.

## 5. Conclusions

In this multicenter, real-world cohort of patients treated with evolocumab following ACS, sustained reductions in LDL-C and total cholesterol were observed over up to 3 years of follow-up. Although patients initiating therapy earlier had higher baseline risk profiles, no statistically significant differences in lipid trajectories or ASCVD outcomes were observed after adjustment. Given the observational design and potential for residual confounding, these findings should be interpreted as descriptive rather than causal. Residual ASCVD risk remained substantial, highlighting the importance of comprehensive secondary prevention strategies. Findings were consistent across both multivariable-adjusted and propensity score-matched analyses, although residual confounding cannot be excluded. Prospective studies with standardized follow-up and time-to-event analyses are needed to better define the relationship between treatment timing and clinical outcomes.

## Figures and Tables

**Table 1:** Baseline Characteristics.

Category	Variable	Value
**Demographics**	Age (years)	53.05 ± 11.62
	Weight (kg)	80.75 ± 14.65
	BMI (kg/m^2^)	29.67 ± 9.52
	Male sex	421 (80.2%)
**Comorbidities/Conditions**	Diabetes mellitus	360 (68.6%)
	Hypertension	381 (72.7%)
	Prior CVD	262 (50.0%)
	Heart failure	140 (26.8%)
	Dyslipidemia	480 (92.0%)
	Chronic kidney disease	42 (8.0%)
	Liver disease	3 (0.6%)
**ASCVD Risk Equivalent**	Familial hypercholesterolemia *	28 (5.4%)
	Family history of premature CVD	28 (5.3%)
	Metabolic syndrome	117 (22.3%)
	Elevated triglycerides (≥150 mg/dL; 1.7 mmol/L)	112 (21.3%)
	Long-standing diabetes mellitus	65 (12.4%)
	LDL ≥ 190 mg/dL; 4.9 mmol/L	68 (13.0%)
	Albuminuria	4 (0.8%)
**Smoking History**	Current smoker	83 (15.8%)
	Former smoker	93 (17.7%)
	Heavy smoker **	45 (8.6%)
	Never smoker	122 (23.2%)
	Unknown smoking status	182 (34.7%)
**Baseline Medications (Prior ACS)**	Aspirin	465 (88.6%)
	Clopidogrel	320 (61.0%)
	Ticagrelor	110 (21.0%)
	Prasugrel	3 (0.6%)
	Anticoagulants	127 (24.2%)
	High-intensity statin ***	361 (68.8%)
	Ezetimibe	71 (13.6%)
	Beta-blocker	456 (86.9%)
	ACE inhibitor	225 (42.9%)
	ARB	150 (28.6%)
	ARNI	59 (11.3%)
	Spironolactone	105 (20.0%)
	Colchicine	13 (2.5%)
	Statin intolerance	25 (4.8%)
**Statin use during follow-up**	Yes	466 (88.8%)
	No	57 (10.9%)
	Missing	2 (0.4%)
**Ezetimibe during follow-up**	Yes	222 (42.3%)
	No	303 (57.7%)

* Familial hypercholesterolemia was defined as a documented clinical or genetic diagnosis in the medical record and was not inferred solely from LDL-C levels. ** Smokes 20 or more cigarettes a day. *** High-intensity statin: 40–80 mg of atorvastatin or 20–40 mg rosuvastatin.

**Table 2:** Lipid Response Across Follow-up Time Points.

Lipid Parameter	Time Point	n	Mean (mmol/L)	SD
**TC**	Baseline	525	5.20	1.67
	3 months	78	3.37	1.26
	6 months	92	3.51	1.70
	1 year	107	3.47	1.34
	2 years	67	3.91	1.56
	3 years	36	3.79	1.14
**LDL-C**	Baseline	525	3.68	1.66
	3 months	77	1.83	1.16
	6 months	90	2.02	1.62
	1 year	107	2.00	1.34
	2 years	67	2.29	1.41
	3 years	36	2.01	1.03
**TG**	Baseline	525	2.12	1.46
	3 months	76	1.80	1.35
	6 months	92	1.70	1.38
	1 year	107	1.64	0.95
	2 years	67	1.74	1.06
	3 years	35	2.13	1.17
**HDL-C**	Baseline	525	0.99	0.27
	3 months	76	1.01	0.28
	6 months	86	1.01	0.25
	1 year	103	1.00	0.27
	2 years	66	1.00	0.25
	3 years	36	0.96	0.23

Values are presented as mean ± standard deviation. Analyses were conducted using an available-case approach; therefore, the number of patients (n) varies across time points. Each time point includes partially overlapping patient subsets and does not represent a fixed longitudinal cohort.

**Table 3:** Changes in Lipid Markers from Baseline Prior to Recurrent ASCVD Events.

Marker	Time	N	Mean Difference	SD	95% CI	*p*-Value
**TC**	3 months	77	–1.73	1.95	–2.18, –1.29	<0.001
	6 months	91	–1.55	1.55	–1.87, –1.22	<0.001
	1 year	106	–1.54	1.79	–1.88, –1.19	<0.001
	2 years	66	–1.32	2.21	–1.86, –0.78	<0.001
	3 years	36	–1.11	1.54	–1.63, –0.59	<0.001
**LDL-C**	3 months	76	–1.57	1.72	–1.96, –1.17	<0.001
	6 months	89	–1.41	1.48	–1.72, –1.10	<0.001
	1 year	106	–1.50	1.79	–1.84, –1.15	<0.001
	2 years	65	–1.42	2.54	–2.05, –0.79	<0.001
	3 years	35	–1.10	1.28	–1.54, –0.66	<0.001
**TG**	3 months	75	–0.491	1.91	–0.93, –0.05	0.029
	6 months	90	–0.466	1.17	–0.71, –0.22	<0.001
	1 year	106	–0.483	1.24	–0.72, –0.24	<0.001
	2 years	66	–0.185	1.21	–0.48, 0.11	0.218
	3 years	35	–0.247	1.28	–0.69, 0.19	0.264
**HDL-C**	3 months	73	+0.005	0.21	–0.04, 0.05	0.846
	6 months	83	–0.005	0.21	–0.05, 0.04	0.812
	1 year	101	+0.011	0.20	–0.03, 0.05	0.573
	2 years	65	+0.035	0.22	–0.02, 0.09	0.201
	3 years	36	–0.071	0.23	–0.15, 0.01	0.076

**Table 4:** Comparison of Baseline Characteristics Between Early vs. Late Evolocumab Initiation.

Category	Variable	Late Initiation (n = 356)	Early Initiation (n = 155)	*p*-Value
**Demographics**	Age (years), mean ± SD	52.34 ± 11.07	54.00 ± 12.44	0.135
	Weight (kg), mean ± SD	80.82 ± 15.25	80.56 ± 13.37	0.851
	BMI (kg/m^2^), mean ± SD	29.67 ± 8.48	29.73 ± 11.83	0.947
	Male sex	288 (80.9%)	122 (78.7%)	0.652
**Comorbidities**	Diabetes mellitus	262 (73.6%)	87 (56.1%)	<0.001
	Hypertension	275 (77.2%)	97 (63.0%)	0.001
	Familial hypercholesterolemia	16 (4.5%)	11 (7.1%)	0.324
	Family history of ASCVD	19 (5.3%)	9 (5.8%)	0.998
	Liver disease	1 (0.3%)	2 (1.3%)	0.450
	Prior ASCVD	173 (48.7%)	82 (52.9%)	0.441
	Heart failure	89 (25.1%)	47 (30.5%)	0.243
**Smoking History**	Current smoker	56 (15.7%)	26 (16.8%)	0.665
	Former smoker	67 (18.8%)	25 (16.1%)	—
	Heavy smoker (>1 pack/day)	26 (7.3%)	17 (11.0%)	—
	Never smoker	82 (23.0%)	36 (23.2%)	—
**ASCVD Risk-Equivalent Conditions**	Elevated CRP	6 (1.7%)	2 (1.3%)	1.000
	Chronic kidney disease	28 (7.9%)	13 (8.4%)	0.982
	Chronic inflammatory disease	8 (2.2%)	2 (1.3%)	0.711
	Elevated triglycerides	90 (25.3%)	22 (14.2%)	0.008
	Metabolic syndrome	101 (28.4%)	16 (10.3%)	<0.001
	LDL ≥ 190 mg/dL	45 (12.6%)	23 (14.8%)	0.595
	Long-standing diabetes	57 (16.0%)	8 (5.2%)	0.001
	Albuminuria	4 (1.1%)	0 (0.0%)	0.436
**Laboratory Parameters**	TC (mmol/L)	5.03 ± 1.60	5.61 ± 1.71	<0.001
	LDL-C (mmol/L)	3.51 ± 1.61	4.08 ± 1.68	<0.001
	HDL-C (mmol/L)	0.99 ± 0.27	1.00 ± 0.27	0.665
	TG (mmol/L)	2.10 ± 1.28	2.20 ± 1.81	0.461
	Hemoglobin A1c (%)	7.88 ± 2.21	7.80 ± 2.39	0.728
**Statin use during follow-up**	Yes	336 (94.6%)	124 (80.0%)	<0.001
	No	19 (5.4%)	31 (20.0%)	
**Statin intensity**	High-intensity	219 (61.5%)	88 (56.8%)	0.006
	Moderate-intensity	98 (27.5%)	34 (21.9%)	
	Low-intensity	5 (1.4%)	1 (0.6%)	
	Unknown	34 (9.6%)	32 (20.6%)	
**Ezetimibe use**	Yes	160 (44.9%)	60 (38.7%)	0.226
	No	196 (55.1%)	95 (61.3%)	

Values are presented as mean ± standard deviation for continuous variables and number (percentage) for categorical variables. *p*-values were calculated using independent *t*-tests for continuous variables and χ^2^ tests for categorical variables. Early initiation was defined as initiation of evolocumab ≤ 1 month after the index ACS event, and late initiation was defined as initiation > 1 month after the index ACS event. Differences between groups reflect baseline imbalances and may indicate confounding by indication.

**Table 5:** Comparison of Lipid and ASCVD Outcomes Between Early vs. Late Evolocumab Initiation.

Category	Variable	Late Initiation (n = 356)	Early Initiation (n = 155)	*p*-Value
**Lipid Parameters**	TC, 3 months	3.48 ± 1.30	2.99 ± 1.13	0.140
	TC, 6 months	3.61 ± 1.78	3.11 ± 1.39	0.243
	TC, 1 year	3.53 ± 1.40	3.26 ± 1.16	0.414
	TC, 2 years	3.71 ± 1.59	4.28 ± 1.31	0.180
	TC, 3 years	3.80 ± 1.18	3.67 ± 0.92	0.836
	LDL-C, 3 months	1.89 ± 1.21	1.64 ± 1.00	0.436
	LDL-C, 6 months	2.03 ± 1.57	1.95 ± 1.83	0.847
	LDL-C, 1 year	2.05 ± 1.39	1.81 ± 1.12	0.453
	LDL-C, 2 years	2.13 ± 1.43	2.56 ± 1.16	0.258
	LDL-C, 3 years	2.03 ± 1.07	1.89 ± 0.78	0.808
	HDL-C, 3 months	1.01 ± 0.29	1.02 ± 0.28	0.915
	HDL-C, 6 months	1.03 ± 0.24	0.97 ± 0.26	0.324
	HDL-C, 1 year	1.02 ± 0.28	0.97 ± 0.26	0.441
	HDL-C, 2 years	0.99 ± 0.24	1.04 ± 0.29	0.465
	HDL-C, 3 years	0.95 ± 0.23	1.00 ± 0.27	0.679
	TG, 3 months	1.87 ± 1.28	1.63 ± 1.62	0.526
	TG, 6 months	1.81 ± 1.52	1.31 ± 0.70	0.148
	TG, 1 year	1.63 ± 0.98	1.69 ± 0.87	0.787
	TG, 2 years	1.75 ± 1.15	1.71 ± 0.85	0.875
	TG, 3 years	2.14 ± 1.18	2.09 ± 1.24	0.935
**Recurrent ASCVD Events**	Any recurrent ASCVD event	47 (13.2%)	28 (18.4%)	0.171
	Adjusted OR (Early vs. Late)	Reference	OR 1.50 (95% CI 0.82–2.70)	0.170
	Repeat coronary revascularization	22 (11.1%)	14 (17.1%)	0.240
	Any second recurrent ASCVD event	12 (3.8%)	8 (6.2%)	0.385
**Hospitalizations**	No ASCVD-related hospitalization	135 (38.4%)	32 (20.9%)	<0.001
	1 ASCVD-related hospitalization	169 (48.2%)	83 (54.2%)	0.287
	≥2 ASCVD-related hospitalizations	48 (13.4%)	38 (25.5%)	<0.001
**Mortality**	In-hospital death	7 (2.0%)	4 (2.6%)	0.916

Abbreviations: TC, total cholesterol; LDL-C, low-density lipoprotein cholesterol; HDL-C, high-density lipoprotein cholesterol; TG, triglycerides; ASCVD, atherosclerotic cardiovascular disease; OR, odds ratio; CI, confidence interval; SD, standard deviation.

**Table 6:** Linear Regression Results for Lipid, Hospitalization, and Recurrent ASCVD Count Outcomes in the Propensity Score-Matched Cohort.

Time Point	Outcome	Early Initiation Mean (SD)	Late Initiation Mean (SD)	β (95% CI)	*p*-Value
**3 Months**	TC	3.33 (0.77)	3.06 (0.64)	0.272 (−0.575, 1.119)	0.500
	LDL-C	1.80 (0.79)	1.59 (0.73)	0.216 (−0.832, 1.264)	0.661
	HDL-C	1.16 (0.32)	0.92 (0.29)	0.240 (−0.135, 0.615)	0.191
	TG	2.49 (3.21)	1.53 (1.07)	0.958 (−1.430, 3.346)	0.399
**6 Months**	TC	2.73 (1.14)	3.85 (2.06)	−1.119 (−2.661, 0.423)	0.145
	LDL-C	1.25 (0.92)	2.30 (2.06)	−1.048 (−2.532, 0.435)	0.156
	HDL-C	0.91 (0.22)	0.96 (0.33)	−0.054 (−0.314, 0.206)	0.669
	TG	1.43 (0.71)	1.95 (2.44)	−0.516 (−2.193, 1.160)	0.527
**1 Year**	TC	3.19 (1.25)	3.52 (1.28)	−0.335 (−1.408, 0.739)	0.525
	LDL-C	1.62 (1.17)	1.98 (1.40)	−0.355 (−1.445, 0.735)	0.507
	HDL-C	1.00 (0.26)	1.13 (0.30)	−0.130 (−0.369, 0.108)	0.268
	TG	1.50 (0.58)	1.29 (0.51)	0.213 (−0.255, 0.681)	0.356
**2 Years**	TC	4.60 (1.30)	3.49 (1.23)	1.115 (−0.010, 2.241)	0.052
	LDL-C	2.86 (1.06)	1.99 (1.38)	0.875 (−0.223, 1.973)	0.112
	HDL-C	0.99 (0.35)	1.00 (0.22)	−0.008 (−0.272, 0.255)	0.948
	TG	2.02 (0.96)	1.38 (0.63)	0.634 (−0.089, 1.357)	0.083
**Count Outcomes**	No. of ASCVD-related hospitalizations	1.31 (1.22)	0.99 (0.83)	0.325 (−0.001, 0.651)	0.051
	No. of recurrent ASCVD events	0.43 (1.10)	0.58 (1.65)	−0.151 (−0.667, 0.364)	0.562

Abbreviations: TC, total cholesterol; LDL-C, low-density lipoprotein cholesterol; HDL-C, high-density lipoprotein cholesterol; TG, triglycerides; β, regression coefficient; CI, confidence interval; SD, standard deviation; ASCVD, atherosclerotic cardiovascular disease.

**Table 7:** Logistic Regression Results for Clinical Outcomes in the Propensity Score-Matched Cohort.

Outcome	Early Initiation Events (%)	Late Initiation Events (%)	OR (95% CI)	*p*-Value
Any recurrent ASCVD event	15 (18.8%)	12 (15.0%)	1.308 (0.570–3.054)	0.527
Second recurrent ASCVD event	7 (11.1%)	5 (7.4%)	1.575 (0.476–5.585)	0.459
Third recurrent ASCVD event	4 (6.5%)	3 (4.5%)	1.448 (0.307–7.612)	0.637

Abbreviations: OR, odds ratio; CI, confidence interval; ASCVD, atherosclerotic cardiovascular disease.

## Data Availability

The data presented in this study are available on request from the corresponding author due to institutional and ethical restrictions related to patient confidentiality.

## Abbreviations

The following abbreviations are used in this manuscript:

ACS	Acute coronary syndrome
ASCVD	Atherosclerotic cardiovascular disease
BMI	Body mass index
CI	Confidence interval
CKD	Chronic kidney disease
CRP	C-reactive protein
HDL-C	High-density lipoprotein cholesterol
HF	Heart failure
IRB	Institutional Review Board
LDL-C	Low-density lipoprotein cholesterol
LLT	Lipid-lowering therapy
NSTEMI	Non-ST-segment elevation myocardial infarction
OR	Odds ratio
PCSK9	Proprotein convertase subtilisin/kexin type 9
SD	Standard deviation
STEMI	ST-segment elevation myocardial infarction
TC	Total cholesterol
TG	Triglycerides
